# Postoperative computed tomography of insufflated lung specimens obtained by video-assisted thoracic surgery: detection and margin assessment of pulmonary nodules

**DOI:** 10.1590/0100-3984.2021.0046

**Published:** 2022

**Authors:** Milene da Silva Antunes, Bruno Hochhegger, Giordano Rafael Tronco Alves, Fernando Ferreira Gazzoni, Gabriele Carra Forte, Rubens Gabriel Feijó Andrade, José Carlos Felicetti

**Affiliations:** 1 Universidade Federal de Ciências da Saúde de Porto Alegre (UFCSPA), Porto Alegre, RS, Brazil.; 2 Pontifícia Universidade Católica do Rio Grande do Sul (PUCRS), Porto Alegre, RS, Brazil.; 3 Division of Imaging Diagnosis, Hospital Santa Casa de Uruguaiana, Uruguaiana, RS, Brazil.

**Keywords:** Tomography, X-ray computed, Thoracic surgery, video-assisted, Lung neoplasms/diagnostic imaging, Tomografia computadorizada, Cirurgia torácica videoassistida, Neoplasias pulmonares/diagnóstico por imagem

## Abstract

**Objective:**

To investigate the utility of computed tomography (CT) scans to detect and assess the margin status of pulmonary nodules that were insufflated after being resected by video-assisted thoracic surgery.

**Materials and Methods:**

This was a novel multicenter study conducted at two national referral centers for thoracic diseases. Patients suspected of having lung cancer underwent video-assisted thoracic surgery for the resection of pulmonary nodules, which were submitted to postoperative CT. Measurements from the CT scans were compared with the results of the histopathological analysis.

**Results:**

A total of 37 pulmonary nodules from 37 patients were evaluated. The mean age of the patients was 65 years (range, 36-84 years), and 27 (73%) were female. A CT analysis of insufflated specimens identified all 37 nodules, and 33 of those nodules were found to have tumor-free margins. The histopathological analysis revealed lung cancer in 30 of the nodules, all with tumor-free margins, and benign lesions in the seven remaining nodules.

**Conclusion:**

Postoperative CT of insufflated suspicious lung lesions provides real-time detection of pulmonary nodules and satisfactory assessment of tumor margins. This initial study shows that CT of insufflated lung lesions can be a valuable tool at centers where intraoperative histopathological analysis is unavailable.

## Abstract (pt)

**Objetivo:**

Investigar a utilidade da tomografia computadorizada (TC) para a detecção e avaliação de margens de nódulos pulmonares que foram insuflados após ressecção por cirurgia torácica videoassistida.

**Materiais e Métodos:**

Um inédito estudo multicêntrico foi conduzido em dois centros de referência nacional para doenças torácicas. Nódulos foram ressecados por cirurgia torácica videoassistida de pacientes com suspeita de câncer de pulmão e submetidos a TC pós-operatória. As medidas radiológicas da TC foram comparadas com as da análise patológica.

**Resultados:**

Um total de 37 pacientes foi avaliado. A idade média foi de 65 anos (variação: 36-84 anos) e 27 indivíduos (73%) eram do sexo feminino. A análise por TC dos espécimes insuflados identificou todas as 37 lesões e 33 delas com margens livres. A análise patológica revelou 30 casos de câncer de pulmão, todos com margens livres, e sete lesões não malignas.

**Conclusão:**

A TC pós-operatória de lesões pulmonares insufladas com suspeita de malignidade provê detecção em tempo real de nódulos pulmonares e aceitável avaliação de margens tumorais. Este estudo inicial demonstra que a TC de lesões pulmonares insufladas pode ser uma ferramenta valiosa em centros em que a análise histopatológica intraoperatória é indisponível.

## INTRODUCTION

Recent advances in computed tomography (CT) of the chest have provided more accurate detection of pulmonary nodules, which are defined as intraparenchymal lesions smaller than 3 cm at their greatest diameter^(1-3)^. The management of pulmonary nodules can be challenging, not only in economic terms but also in regard to patient distress. The latter is particularly relevant for those with a history of cancer^(4)^.

Although the majority of pulmonary nodules are secondary to benign or inflammatory changes, a smaller yet significant proportion are malignant^(3)^. Unfortunately, fine needle aspiration biopsy has demonstrated poor performance for the diagnosis of such lesions^(5)^. More recently, video-assisted thoracic surgery (VATS) has been shown to be relatively free of the sampling errors associated with fine needle aspiration biopsy and has therefore been widely used in order to identify and treat pulmonary nodules^(1,6)^. Consequently, VATS is currently considered the gold-standard, because it is a cost-effective technique that can achieve results similar to those of open thoracotomy, with fewer complications^(7,8)^. However, in a previous study of 92 consecutive patients undergoing VATS^(9)^, conversion to open thoracotomy was necessary in 50 (54.3%). In that study, the most common reason for conversion to open thoracotomy was failure to locate the nodules. The authors conducted univariate and multivariate analyses of the 11 variables examined. They found that if the distance from the pleural surface to the nodule edge was greater than 5 mm, the probability of failure to detect a nodule was 63%^(9)^.

Data regarding nodule resection and tumor-free margins are crucial for determining therapeutic and follow-up interventions^(10)^. During histopathological examination, however, lung deflation and formalin use can lead to shrinkage effects, which present difficulties for pathologists in differentiating collapsed healthy lungs from small foci of carcinomas^(11)^. Consequently, some authors have proposed an inflation technique to preserve specimen morphology^(12,13)^.

The aim of the present study was to investigate the utility of CT scans for real-time detection of pulmonary lesions and for the assessment of tumor-free margins in insufflated pulmonary nodule specimens obtained by VATS resection.

## MATERIALS AND METHODS

### Study design

This was a multicenter study conducted at two national referral centers for thoracic diseases between June 2019 and October 2020. This study followed the recommendations of the Declaration of Helsinki, and the study protocol was approved by the local research ethics committee. All of the researchers signed a confidentiality agreement to ensure the anonymity of the data obtained.

VATS-resected nodules were prospectively included from patients who were suspected of having lung cancer. Patients who had previously undergone lung surgery were excluded, as were those receiving chemotherapy or radiotherapy. After applying an inflation technique to the nodule samples and obtaining CT scans, we compared the radiological measurements with those obtained from histopathology.

The following variables were extracted from the final histopathology report for each sample: patient age; patient sex; and histologic characteristics of the tumor (e.g., size, location, and tumor-free margins).

### VATS

Prior to surgery, nodules were marked with methylene blue tattooing^(6,7)^. In brief, the dye was injected immediately adjacent to the nodule and along the needle tract up to the pleural surface as the needle was retracted, thereby enabling thoracoscopic guidance. The VATS was performed by creating three ports and using endovascular gastrointestinal anastomosis staplers: Endopath (Ethicon Endo-Surgery, Cincinnati, OH, USA) or Endo GIA (AutoSuture, Norwalk, CT, USA). Each surgical specimen was extracted with a specimen retrieval system (EndoBag; AutoSuture). Each collected specimen was subsequently injected with 10 L/min O_2_ from an 18-gauge needle through a segmental bronchus until the specimen was visually insufflated. The CT study and corresponding analysis were completed within 30 min after pulmonary resection and prior to formalin fixation.

### CT scans

The CT scans were acquired in one of two commercially available multidetector (64-slice) scanners: Somatom Sensation 64 (Siemens Medical Systems, Forchheim, Germany); or LightSpeed VCT (GE Healthcare, Milwaukee, WI, USA). The parameters used included the following: collimation, 1 mm; rotation time, 0.33 s; pitch, 1.3; dose, 120 kV and 200 mAs. All of the CT images were reconstructed with 1-mm axial slices. The use of automatic exposure control and soft tissue (standard) kernel was allowed. A data matrix of 512 × 512 pixels was used. The scanners were calibrated periodically according to the manufacturers’ recommendations. Images were evaluated by two thoracic radiologists, each with more than 10 years of experience, and disagreements were resolved by consensus. Compromised margins were defined as those for which the arch distance-to-maximum tumor diameter ratio was greater than 0.9^(14)^.

### Histopathological analysis

Two pathologists, each with more than five years of experience in thoracic diseases, assessed the resected lesions. Specimens were analyzed according to the recommended criteria for examining specimens from patients with primary non-small cell carcinoma, small cell carcinoma, or carcinoid tumor of the lung^(15)^. Tumor-free margins were defined by the presence of uninvolved tissue surrounding a nodule. Margin distance was not recorded, given that previous studies have suggested that this parameter does not influence recurrence or survival^(16)^.

### Statistical analysis

Excel software (Microsoft Corp., Redmond, WA, USA) was used for data tabulation and descriptive analyses. Continuous variables are expressed as means, medians, ranges, and standard deviations, whereas categorical variables are expressed as absolute and relative frequencies.

## RESULTS

Thirty-seven patients with pulmonary nodules were examined. The mean age was 65 years (range, 36-84 years), and 27 (73%) of the patients were female. The mean pulmonary nodule diameter was 1.3 cm (range, 0.6-2.0 cm). Of the 37 nodules evaluated, 17 (46.0%) were located in the right upper lobe, nine (24.3%) were located in the right lower lobe, eight (21.6%) were located in the left upper lobe, and three (8.1%) were located in the left lower lobe. Table 1 summarizes those findings.

**Table 1:** Characteristics of the patients and pulmonary nodules.

Characteristic	(N = 37)
Age (years), mean (range)	65 (36-84)
Sex, n (%)	
Male	10 (27.0)
Female	27 (73.0)
Nodule size (cm), mean (range)	1.3 (0.6-2.0)
Nodule location, n (%)	
Right upper lobe	17 (46.0)
Right lower lobe	9 (24.3)
Left upper lobe	8 (21.6)
Left lower lobe	3 (8.1)

The CT analysis of the insufflated specimens identified all 37 pulmonary lesions, 33 of which were found to have tumor-free margins. Histopathological analysis of the same specimens showed that 30 were malignant nodules, all of which had been resected with tumor-free margins. The remaining seven lesions were classified as benign (Table 2).

**Table 2:** Comparison between benign and malignant nodules in terms of patient demographic data, CT findings, and nodule size.

Variable	Histopathological classification		
	Benign (n = 7)	Malignant (n = 30)	*P*-value
Sex, n (%)			
Female	4 (57.1)	23 (76.7)	0.273
Male	3 (42.9)	7 (23.3)	
Age (years), mean ± SD	66.7 ± 5.2	64.4 ± 11.2	0.197
CT classification of margins, n (%)			
Tumor-free	5 (71.4)	28 (93.3)	0.388
Compromised	2 (28.6)	2 (6.7)	
Nodule size (cm), median (IQR)	1 (0.7-2.0)	0.9 (0.6-1.9)	0.937

IQR, interquartile range.

**Figure 1:**
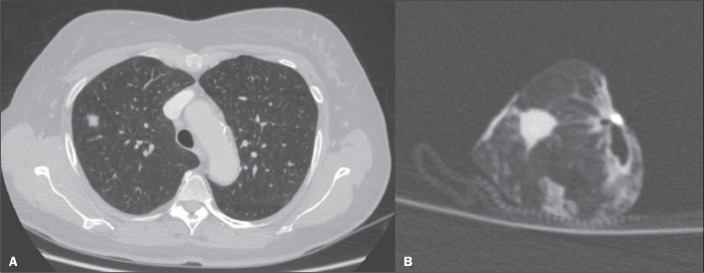
A: Axial CT scan of a 58-year-old female patient, showing a solid pulmonary nodule in the right upper lobe. B: A corresponding CT scan of the insufflated specimen obtained by VATS segmentectomy detected the nodule and demonstrated tumor-free margins. The histopathological analysis confirmed the tumor-free margins.

**Figure 2:**
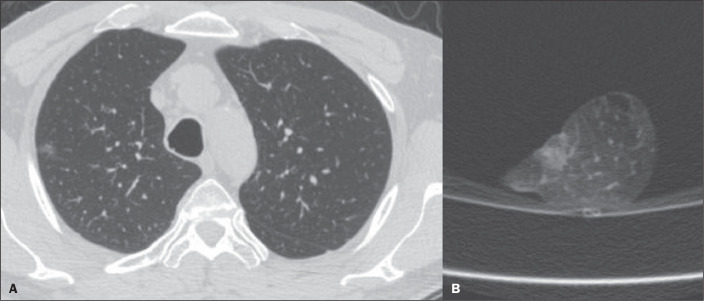
A: Axial CT scan of a 41-year-old male patient revealed a subsolid pulmonary nodule in the right upper lobe. B: CT scan of the corresponding insufflated specimen, suggesting that the margins were compromised. However, the histopathological analysis confirmed tumor-free margins.

The correlation between CT and the histopathology was very good (Figure 1). Among the four nodules classified as having compromised margins on CT, the histopathology showed tumor-free margins (Figure 2) in two, a focal area of subpleural fibrosis in one, and an area of chronic nonspecific granulomatous inflammation in one. All of the treatments administered in our sample were determined on the basis of the histopathology results.

## DISCUSSION

Recent studies conducted in Brazil have highlighted the importance of imaging examinations, especially CT, to improving the diagnosis of pulmonary diseases^(17-21)^. To our knowledge, this is the first study to investigate insufflated specimens by postoperative CT to identify nodules and predict tumor-free margins, with subsequent histopathology correlation. Since its development in the early 1990s, VATS has become a state-of-the-art method for accurately resecting pulmonary nodules. The use of this procedure has reduced the duration of hospital stays, patient discomfort, and overall costs in comparison with open thoracotomy^(7)^. The use of VATS in combination with methylene blue tattooing made it possible to localize small nodules, even those that were not palpable.

Overall, we observed a strong association between postoperative CT of the insufflated lesions and histopathology, with only two discrepant reports in the malignant group. Those two reports were related to two cases of lepidic-growth carcinomas, which may undergo shrinkage of their lepidic component upon histopathology examination, as previously described by Isaka et al.^(12)^. We anticipate that the positive predictive value of this technique for tumor-free margins will make it useful as a rapid and accurate assessment of margins in clinical practice.

Granulomatous diseases are known pose challenges during the workup of suspected lung cancer cases^(3,9)^. In the present study, a relatively high proportion of the specimens (19%) were benign, those specimens including nodules found to present various fibrotic changes, alveolar proteinosis, and lymphocytic pneumonia. That finding may be due to the geographic region, which is endemic for granulomatous diseases^(22)^.

Our study has some limitations. First, due to the novel character of the study, the patient sample was small. Second, specific parameters were not stipulated for specimen insufflation, because lesions can vary in volume, and that could limit the replicability. Third, the adoption of other CT criteria^(23)^, such as contact with the pleural surface greater than 3 cm, pleural thickening, and an obtuse angle between the nodule and pleural surface, could have yielded small differences between the imaging and histopathological analyses.

In conclusion, postoperative CT of insufflated specimens detected all pulmonary lesions and exhibited a strong overall correlation with histopathology. This initial study indicates that CT can identify pulmonary nodules and assess tumor-free margins, demonstrating its potential applicability in real-time, intraoperative settings.
